# Firefighters’ occupational stress and its correlations with cardiorespiratory fitness, arterial stiffness, heart rate variability, and sleep quality

**DOI:** 10.1371/journal.pone.0226739

**Published:** 2019-12-23

**Authors:** Young-Sook Yook

**Affiliations:** Department of Exercise Rehabilitation Welfare, Sungshin Women’s University, Seoul, Republic of Korea; Ritsumeikan University, JAPAN

## Abstract

This study investigated the correlations between firefighters’ occupational stress and cardiorespiratory fitness, arterial stiffness, heart rate variability, and sleep quality. We examined 705 male firefighters aged 40–50 years in Seoul City, Korea from November 2016–December 2017. The Occupational stress scale was used to evaluate occupational stress; an exercise stress test was administered to measure participants’ maximal oxygen uptake (VO_2_max); brachial-ankle pulse wave velocity was used to measure firefighters’ arterial stiffness; their autonomic nervous system activities were analyzed to determine heart rate variability (HRR); and the Pittsburgh Sleep Quality Index was used to assess their sleep quality. We divided the sample population into tertile groups per their occupational stress scores; i.e., low-stress group (n = 233), medium-stress group (n = 237), and high-stress group (n = 235). They were compared per each indicator and correlations were examined. There was a significant difference in VO_2_max (p < .01), and arterial stiffness (p < .001) according to occupational stress levels. Occupational stress was significantly correlated with cardiorespiratory fitness (r = -.820, p < .05), arterial stiffness (r = .085, p < .05), and sleep quality (r = .276, p < .001), but not HRR. In conclusion, Firefighters’ occupational stress is a key factor behind their elevated risk of cardiovascular diseases; therefore, we recommend programs aimed at reducing their occupational stress and preventing cardiovascular diseases.

## Introduction

Firefighters save human lives and provide relief in various emergency situations including natural calamities, industrial accidents, and human-caused disasters. They encounter many precarious, life-or-death situations while on duty and are exposed to wide-ranging serious dangers including heat, chemical hazards, biological hazards, physical strain, and mental stress [1]. Firefighting is thus a high-risk job that involves an elevated threat of cardiovascular diseases induced by job-related stress [2, 3, 4].

Previous studies have found that occupational stress is associated with cardiovascular diseases and mortality risk [5]; however, prior literature has also reported a reduced risk in participants with higher fitness levels [6, 7]. These findings highlight the importance of exercises aimed at enhancing physical fitness in preventing cardiovascular diseases induced by occupational stress.

Occupational stress can also cause cardiovascular diseases by accelerating arterial stiffening. Recent studies reported that the pulse wave velocity (PWV), an indicator of arterial stiffness, can serve as a meaningful, independent predictor for cardiovascular mortality [8, 9, 10]. Another important mechanism behind the link between occupational stress and cardiovascular diseases may involve stress-induced activation of the hypothalamic-pituitary-adrenal (HPA) axis, which reduces autonomic nervous functions [11]. Heart rate variability (HRR) has also recently emerged as a predictor of cardiovascular diseases according to the physical stress.

Analysis of HRR evaluates the balance between sympathetic and parasympathetic functions within the autonomic nervous system, and stress-induced impairment of autonomic nervous functions reduces heart rate variability [12]. Several studies reported an association between occupational stress and HRR [13, 14, 15].

Recent studies also noted that higher occupational stress in shift workers is associated with decreased sleep quality [16, 17, 18]. Firefighters typically participate in a three-shift working schedule, giving rise to sleep disorders that adversely impact the risk of cardiovascular disease [19]. In a survey of 37,093 firefighters, Korea’s National Emergency Management Agency reported that 36.6% of them require sleep disorder management and 21.9% require treatment [20]. Notably, sleep disorders are associated with the onset of cardiovascular diseases [21].

These considerations warrant analysis of the correlations between firefighters’ occupational stress and the above-mentioned four indicators of cardiovascular diseases—cardiorespiratory fitness, arterial stiffness, HRR, and sleep quality—using appropriate assessment tools to prevent cardiovascular diseases in firefighters. However, a paucity of research has validated the correlation between occupational stress in firefighters with these four factors despite their salience as indicators of cardiovascular diseases. Consequently, we examined the correlations between firefighters’ occupational stress and these cardiovascular disease risk factors.

## Materials and methods

### Participants

Participants were 705 male firefighters aged 40–50 years who visited the National Fitness Center in Seoul, Korea, between November 2016 and December 2017. Participants completed tests concerning body composition, exercise stress, arterial stiffness, heart rate variability. They were also administered surveys on occupational stress and sleep quality. After explaining the contents of the questionnaire they were asked to fill out from themselves. This study was conducted with verbal consent. This study was approved by the Institutional Review Board of Sungshin Women’s University (no. 2018–003).

### Measures

#### Body composition test

Participants’ height and weight were measured using an automatic body measurement device (SH-9600A, Korea). Body mass index (BMI) was calculated using height and weight data: [(weight (kg)/height (m)^2^].

#### Heart rate and blood pressure measurement

Participants’ resting heart rate was measured using an automatic 12-lead standard electrocardiograph (Cardiocare, Korea), and their systolic and diastolic blood pressure were measured using an automatic sphygmomanometer (Cardiocare, Korea).

#### Exercise stress test

Participants’ maximum rate of oxygen consumption (VO_2_max) data were obtained using exercise stress test equipment (Quiton, USA) and an automatic gas analysis system (Jaeger, Germany). The modified Balke protocol was adopted for the test. The test was discontinued when participants met two or more of the to five following criteria: they reached the age-predicted maximum heart rate (220-age), respiratory exchange ratio ≥ 1.15, perceived exertion ≥ 17, a higher exercise load did not increase the oxygen consumption rate, and the participant voluntarily expressed his wish to stop the test.

#### Occupational stress

Participants’ occupational stress was evaluated using the Korean Occupational Stress Scale [22]. This scale comprises eight sub-factors: physical environment, job demand, insufficient job control, interpersonal conflict, job insecurity, organizational system, lack of reward, and occupational climate. Items are responded to using a 4-point Likert scale: “not true at all,” “not true,” “true,” and “very true” (1, 2, 3, and 4, respectively). Higher scores indicate higher occupational stress. Cronbach’s α was .85 in this study.

#### Arterial stiffness

Participants’ arterial stiffness was measured using an automatic waveform analyzer (automatic waveform analyzer; VP-1000, Komaki, Japan), through which brachial-ankle PWV readings were obtained. Participants took a minimum five-minute rest before the test. The arterial PWV (m/s) was defined as the distance of two points (m) divided by the pulse wave transit time (s).

#### Heart rate variability

Participants’ mean heart rate, standard deviation of all the normal R-R intervals (SDNN), and root mean square of successive differences between the normal heart beats (RMSSD) were measured using SA-2002E (Medicore Co. Ltd., Korea). Participants took a minimum five-minute rest before the test. The frequency domain was analyzed to obtain the low-frequency power (LF), high-frequency power (HF), and LF/HF ratio. The frequency domain parameters were computed through logarithmic transformation.

#### Sleep quality

Sohn et al.’s [23] Korean version of Buysse et al.’s [24] Pittsburgh Sleep Quality Index (PSQI-K) was used to assess participants’ sleep quality. The PSQI-K contains seven sub-factors: subjective sleep quality, sleep latency, sleep duration, habitual sleep efficiency, sleep disturbances, use of sleeping medication, and daytime dysfunction. Items are responded to using a 4-point Likert scale (0–3: “not true at all,” “not true,” “true,” and “very true” (0, 1, 2, and 3, respectively); therefore, total scores range from 0 to 21, with higher scores indicating lower sleep quality. Scores of 5 or less signify that participants are sleeping well. When developing the sleep quality questionnaire, the Cronbach’s α indicating internal consistency was. 83. The Cronbach’s α of this study was .89.

### Statistical analyses

SPSS 18.0 (Windows) was used for statistical analyses. Participants were divided into low-stress, medium-stress, and high-stress groups regarding their occupational stress scores. A one-way analysis of variance and a post-hoc test were performed to examine variations in participants’ cardiorespiratory fitness, arterial stiffness, HRR, and sleep quality according to occupational stress levels. A correlation analysis was conducted to examine the Pearson correlation correlations between occupational stress with cardiorespiratory fitness, arterial stiffness, HRR, and sleep quality, with participants’ age, exercise, smoking, and drinking statuses set as covariates. The significance level (α) was set at p < .05.

## Results

### Participants’ physical characteristics according to occupational stress levels

The low-stress, medium-stress, and high-stress groups demonstrated no difference in age, height, weight, BMI, resting heart rate, or systolic/diastolic blood pressure. There was also no variation in participants’ years of work, working hours, exercise, smoking, and drinking status according to occupational stress levels (Table 1).

**Table 1:** Physical characteristics according to occupational stress levels.

	Low (n = 233)	Med (n = 237)	High (n = 235)	P-value
Age (years)	55.73 ± 4.31	55.95 ± 3.72	56.22 ± 3.0	.364
Height (cms)	171.18 ± 4.47	170.43 ± 4.74	171.01 ± 4.56	.179
Weight (kgs)	73.52 ± 8.24	73.01 ± 9.42	73.96 ± 8.45	.495
Body mass index (kg/m^2^)	25.08 ± 2.43	25.15 ± 2.70	25.28 ± 2.49	.680
Resting heart rate (beats per min)	69.98 ± 11.47	68.55 ± 10.68	70.53 ± 10.82	.284
Systolic blood pressure (mmHg)	119.36 ± 15.0	118.37 ± 15.76	120.57 ± 16.2	.479
Diastolic blood pressure (mmHg)	90.97 ± 13.77	90.04 ± 13.12	92.33 ± 14.08	.344
Working period (years)	26.04 ± 6.23	25.83 ± 6.23	26.22 ± 5.05	.774
Working hours (per week)	48.45 ± 10.6	50.59 ± 12.80	49.43 ± 13.01	.194
Exercise status (%)	211 (90.6)	210 (88.6)	206 (87.7)	.596
Smoking status (%)	60 (25.8)	57 (24.1)	54 (31.6)	.780
Drinking status (%)	220 (94.4)	210 (88.6)	216 (91.6)	.074
**Occupational stress index**				
Job demand (score)	31.29 ± 14.67	42.33 ± 11.0	47.94 ± 13.47	
Insufficient job control (score)	46.39 ± 14.45	50.70 ± 11.25	57.23 ± 15.09	
Interpersonal conflict (score)	28.56 ± 11.6	35.26 ± 7.01	44.54 ± 15.03	
Job insecurity (score)	17.17 ± 16.41	34.11 ± 11.61	40.21 ± 15.6	
Organizational system (score)	29.11 ± 12.16	36.81 ± 7.19	51.13 ± 16.07	
Lack of reward (score)	29.71 ± 12.06	37.18 ± 7.22	52.01 ± 16.8	
Organizational climate (score)	22.42 ± 13.66	36.78 ± 7.35	47.87 ± 15.51	
Total (score)	28.39 ± 7.45	38.74 ± 1.95	48.60 ± 5.18	

Data shown as Mean±SD

Exercise status: ≥ 30 mins/day for ≥ 3 days/week; smoking status: ≥ 1 cigarettes daily; drinking status: ≥ once/month.

### Comparison of cardiorespiratory fitness, arterial stiffness, heart rate variability, and sleep quality according to occupational stress levels

VO_2_max was significantly higher in the low-stress group than in the medium- or high-stress groups (Table 2). There was no significant difference in the right, left, and mean arterial stiffness according to occupational stress. Of the HRR parameters, no significant difference in MRR, SDNN, RMSSD, LF, HF, or LF/HF ratio according to occupational stress. Regarding sleep quality, there was a significant difference in sleep quality, sleep latency, sleep duration, habitual sleep efficiency, sleep disturbances, use of sleeping medication, daytime dysfunction, and total sleep quality scores according to occupational stress levels.

**Table 2:** Comparison of cardiovascular fitness, pulse wave velocity, heart rate variability, and sleep quality according to occupational stress levels.

	Low^a^ (n = 233)	Med^b^ (n = 237)	High^c^ (n = 235)	P-value	Post-hoc
**VO**_**2**_**max** (ml/kg/min)	38.99 ± 6.36	37.18 ± 6.92	37.13 ± 7.18	.004	a > bc
**PWV**					
Left PWV (cm/sec)	1401.22 ± 261.08	1393.76 ± 228.49	1441.09 ± 252.76	.084	ns
Right PWV (cm/sec)	1407.32 ± 259.74	1412.47 ± 239.81	1454.20 ± 261.76	.091	ns
Mean PWV (cm/sec)	1404.27 ± 257.74	1403.12 ± 231.30	1447.64 ± 254.64	.086	ns
**Heart rate variability**					
Mean heart rate (beat per min)	70.50 ± 9.87	71.09 ± 11.12	71.15 ± 10.95	.766	ns
SDNN (ms)	56.39 ± 56.49	55.73 ± 61.93	58.68 ± 59.09	.851	ns
RMSSD (ms)	28.40 ± 39.22	27.75 ± 41.05	30.94 ± 47.36	.693	ns
Log LF	5.82 ± 1.62	5.73 ± 1.69	5.88 ± 1.63	.622	ns
Log HF	5.47 ± 1.63	5.36 ± 1.72	5.54 ± 1.77	.517	ns
LF/HF ratio	2.02 ± 2.42	1.96 ± 2.00	1.99 ± 2.32	.959	ns
**Sleep quality**					
Subjective sleep quality (score)	1.93 ± 0.72	2.11 ± 0.59	2.31 ± 0.72	< .001	a < b < c
Sleep latency (score)	1.47 ± 0.67	1.52 ± 0.69	1.76 ± 0.74	< .001	ab < c
Sleep duration (score)	0.81 ± 0.93	0.96 ± 0.91	1.08 ± 0.94	.008	ab < c
Habitual sleep efficiency (score)	0.35 ± 0.80	0.49 ± 0.86	0.55 ± 1.00	.038	ab < c
Sleep disturbances (score)	1.88 ± 0.47	2.02 ± 0.41	2.14 ± 0.57	< .001	a < b < c
Use of sleeping medication (score)	1.07 ± 0.39	1.03 ± 0.16	1.18 ± 0.53	< .001	ab < c
Day time dysfunction (score)	1.14 ± 0.40	1.26 ± 0.49	1.51 ± 0.69	< .001	a < b < c
Total (score)	8.28 ± 3.15	9.22 ± 2.91	10.23 ± 3.65	< .001	a < b < c

Data shown as Mean±SD

SDNN: standard deviation of all the normal R-R intervals; RMSSD: root mean square of successive differences between the normal heart beats; Log LF: log transformation of the low frequency component; Log HF: log transformation of the high frequency component; PWV: pulse wave velocity.

### Correlations of occupational stress with cardiorespiratory fitness, arterial stiffness, heart rate variability, and sleep quality

Table 3 illustrates the negative correlation between occupational stress and cardiorespiratory fitness, the positive correlation between occupational stress and arterial stiffness, no correlation between occupational stress and the parameters of HRR, and a positive correlation between occupational stress and sleep quality.

**Table 3:** Correlation coefficients between occupational stress, cardiovascular fitness, pulse wave velocity, heart rate variability, and sleep quality.

		VO_2_max	Mean PWV	Heart rate variability						Sleep quality
				Mean HRT	SDNN	RM SSD	LogLF	LogHF	LF/HF Ratio	
Occupational Stress	r	-.082*	.085*	-.001	.027	.036	.011	.008	.009	.276**
	p	.030	.024	.985	.466	.338	.766	.836	.806	.001

PWV: pulse wave velocity; Mean HRT: mean heart rate; SDNN: standard deviation of all the normal R-R intervals; RMSSD: root mean square of successive differences between the normal heart beats; Log LF: log transformation of the low frequency component: Log HF: log transformation of the high frequency component.

*p < .05

**p < .01.

Adjusted variables: age, exercise, smoking, and alcohol status.

## Discussion

This study investigated the correlations between firefighters’ occupational stress and their cardiorespiratory fitness, arterial stiffness, heart rate variability, and sleep quality. We also observed a significant negative correlation between occupational stress and cardiorespiratory fitness, arterial stiffness, and sleep quality, but not with HRR.

Firefighters perform various duties including fire prevention, fire suppression, rescue, and medical assistance. These tasks, although essential, entail frequent exposure to traumatic events and a high degree of stress while on duty. Previous studies have confirmed that occupational stress is associated with the onset of cardiovascular diseases [25, 26]. In addition, low cardiorespiratory fitness increases the risk of cardiovascular diseases [27, 28]. Our analysis revealed variations in firefighters’ cardiorespiratory fitness levels according to occupational stress, suggesting an association between occupational stress and cardiorespiratory fitness. Consistent with our findings, Yoon et al. [29] reported a significant correlation (r = –0.25) between occupational stress scores and VO_2_max in 30–50 year old office worker. Exercise reduces stress and is useful in enhancing cardiorespiratory fitness [30, 31]. Previous studies reported that higher levels of stress on the job translated into less exercise [32, 33]. However, firefighters who participated in the study regularly participated in the exercise. As a result, it seems to affect cardiorespiratory fitness and psychological stress reduction.

Occupational stress is associated with cardiovascular diseases by accelerating atherosclerosis. PWV offers a useful insight into arterial stiffness [9]. Atherosclerosis stiffens arterial walls and reduces vascular compliance as the condition progresses, leading to a higher PWV. We observed no variation in arterial stiffness measured through the PWV according to firefighters’ occupational stress levels; however, occupational stress and arterial stiffness were correlated. Jeon et al. [34] reported that firefighters with high occupational stress levels were at an elevated risk of showing a high PWV (1.4 m/s or above) compared to their low-stress counterparts. Kong et al. [35] also discovered a correlation between occupational stress and the PWV in manufacturing-sector workers in their 40s and older. Several previous studies reported that a PWV of 1.4 m/s or more leads to higher atherosclerosis risks. Consistent with these findings, we found that firefighters’ PWV was 1.4 m/s or above, ascribed to the impact of their occupational stress on atherosclerosis. However, arterial stiffness mostly involves other varied factors including obesity, hypertension, diabetes, and dyslipidemia. Future studies on the correlation between occupational stress and arterial stiffness should consider these factors [36].

Another mechanism behind occupational stress-induced cardiovascular diseases involves autonomic imbalance. HRR is a quantified representation of autonomic nervous activities. A low HRR implies sympathetic overreaction or parasympathetic underactivity. Of the frequency domains under HRR, LF indicates sympathetic and parasympathetic activities and HF represents parasympathetic activities. Numerous studies reported that patients with high mental stress or post-traumatic impairments demonstrate a high LF and a low HF [37, 38]. Our analysis found no significant in LF, HF, or the LF/HF ratio according to occupational stress in firefighters. We also observed no correlation between occupational stress and HRR. However, contrary to our findings, Vrijkotte et al. [12] reported lower levels of HRR parameters that represent parasympathetic activities in a high-stress group of office workers. Many other previous studies also identified a significant correlation between occupational stress and HRR [13, 14, 15]. Contrary to previous studies, the recent work intensity of firefighters may affect the HRR test results. We think that future research is needed.

The biological mechanism behind stress-induced sleep disorders involves the activation of the HPA axis and autonomic nervous system that, in both cases, causes arousal [39, 40]. Poor sleep quality exacerbates autonomic imbalance and increases cardiovascular disease risks [41], along with other adverse effects such as cognitive impairments (reduced memory and concentration) and diminished work performance induced by daytime sleepiness [42]. Furthermore, stress is reported to be a common cause of insomnia [43]. Our findings demonstrated variations in firefighters’ sleep quality according to occupational stress levels and a significant correlation between occupational stress and sleep quality. Many other previous studies reported a lower sleep quality in shift workers, such as firefighters, who experience a higher level of job-related stress [16, 18, 44]. Both occupational stress and sleep disorders can compromise work efficiency. This warrants programs aimed at improving sleep disorders induced by occupational stress in firefighters.

This study had some limitations. First, we used convenience sampling to recruit participants, which makes it difficult to generalize our findings. We also cannot confirm causal relationships due to the cross-sectional study design. In addition, although we used valid self-reporting questionnaires to measure participants’ occupational stress and sleep quality, over- and underreporting is possible given the inherent limits to subjective surveys. Despite these limitations, we believe that our findings provide basic data for endeavors toward reducing job-related stress and stress-induced cardiovascular disease risks in firefighters.

## Conclusion

We evaluated the correlation between occupational stress and four indicators of cardiorespiratory fitness, arterial stiffness, HRR, and sleep quality in a group of firefighters within one community. We observed a correlation between firefighters’ occupational stress and their cardiorespiratory fitness, arterial stiffness, and sleep quality. These findings suggest that occupational stress is a key factor behind the elevated risk of cardiovascular diseases in firefighters.

## References

[1] Markowitz JS, Gutterman EM, Schwartz S, Link B, Gorman SM. Acute health effects among firefighters exposed to a polyvinyl chloride (PVC) fire. Am J Epidemiol, 1989; 129(5):1023–1031. 10.1093/oxfordjournals.aje.a115206 2705423

[2] Heinrichs M, Wagner D, Schoch W, Soravia LM, Hellhammer DH, Ehlert U. Predicting posttraumatic stress symptoms from pretraumatic risk factors: a 2-year prospective follow-up study in firefighters. Am J Psychiatry, 2005; 162:2276–2286. 10.1176/appi.ajp.162.12.2276 16330591

[3] Kales SN, Soteriades ES, Christophi CA, Christiani DC. Emergency duties and deaths from heart disease among firefighters in the United States. N Engl J Med, 2007; 356(12):1207–1215. 10.1056/NEJMoa060357 17377158

[4] Saijo Y, Ueno T, Hashimoto Y. Post-traumatic stress disorder and job stress among firefighters of urban Japan. Prehosp Disaster Med, 2012; 27:59–63. 10.1017/S1049023X12000222 22591931

[5] Johnson JV, Stewart W, Hall EM, Fredlund P, Theorell T. Long-term psychosocial work environment and cardiovascular mortality among Swedish men. Am J Public Health, 1996; 86:324–331. 10.2105/ajph.86.3.324 8604756 PMC1380510

[6] Hamer M, Molloy GJ, Stamatakis E. Psychological distress as a risk factor for cardiovascular events: pathophysiological and behavioral mechanisms. J Am Coll Cardiol, 2008; 52:2156–2162. 10.1016/j.jacc.2008.08.057 19095133

[7] Holtermann A, Mortensen OS, Burr H, Sogaard K, Gyntelberg F, Suadicani P. Physical work demands, hypertension status, and risk of ischemic heart disease and all-cause mortality in the Copenhagen Male Study. Scand J Work Environ Health, 2010; 36:466–472. 10.5271/sjweh.3120 20852831

[8] Blacher J, Asmar R, Djane S, London GM, Safar ME. Aortic pulse wave velocity as a marker of cardiovascular risk in hypertensive patients. Hypertens, 1999; 33:1111–1117. 10.1161/01.hyp.33.5.1111 10334796

[9] Cortez-Cooper MY, Supak JA, Tanaka H. A new device for automatic measurements of arterial stiffness and anklebrachial index. Am J Cardiol, 2003; 91:1519–1522. 10.1016/s0002-9149(03)00416-8 12804752

[10] Lehmann ED, Riley WA, Clarkson P, Gosling RG. Noninvasive assessment of cardiovascular disease in diabetes mellitus. Lancet, 1997; 350:S14–S19. 10.1016/s0140-6736(97)90023-4 9250278

[11] Hamer M. Psychosocial stress and cardiovascular disease risk: the role of physical activity. Psychosom Med, 2012; 74:896–903. 10.1097/PSY.0b013e31827457f4 23107839

[12] Vrijkotte TGM, van Doornen LJP, de Geus EJC. Effects of work stress on ambulatory blood pressure, heart rate, and heart rate variability. Hypertens, 2000; 35:880–886. 10.1161/01.hyp.35.4.880 10775555

[13] Dishman RK, Nakamura Y, Garcia ME, Thompson RW, Dunn AL, Blair SN. Heart rate variability, trait anxiety, and perceived stress among physically fit men and women. Int J Psychophysiol, 2000: 37;121–133. 10.1016/s0167-8760(00)00085-4 10831999

[14] Kageyama T, Nishikido N, Kobayashi T, Kurokawa Y, Kaneko T, Kabuto M. Long commuting time, extensive over time, and sympathodominant state assessed in terms of short-term heart rate variability among male white-collar workers in the Tokyo megalopolis. Ind Health, 1998; 36:209–217. 10.2486/indhealth.36.209 9701898

[15] van Amelsvoort LGPM Schouten EG, Maan AC, Swenne CA, Kok FJ. Occupational determinants of heart rate variability. Int Arch Occup Environ Health, 2000; 73;255–262. 10.1007/s004200050425 10877031

[16] Akerstedt T. Shift work and disturbed sleep/wakefulness. J of Occup Med, 2003; 53(1):89–94. 10.1093/occmed/kqg046 12637592

[17] Kim YG, Yoon DY, Kim JI, Chea CH, Hong YS, Yang CG, et al Effect of health on shift work: general and psychological health, sleep, stress, quality of life. Korean J Occup Environ Med, 2002; 14(3):247–256.

[18] Nasermoaddeli A, Sekine M, Hamanishi S. Kagamimori S. Job strain and sleep quality in Japanese civil servants with special reference to sense of coherence, J Occup Health, 2002; 44(1):337–342. 10.1539/joh.44.337

[19] Parish J. Sleep-related problems in common medical conditions. Chest, 2009; 35:563–572. 10.1378/chest.08-0934 19201722

[20] National Emergency Management Agency. National firefighters personnel psychological assessment questionnaire analysis. National Emergency Management Agency 2014.

[21] Garbarino S, Guglielmi O, Sanna A, Mancardi GL, Magnavita N. Risk of occupational accidents in workers with obstructive sleep apnea: systematic review and meta-analysis. Sleep, 2016, 39:1211–1218. 10.5665/sleep.5834 26951401 PMC4863208

[22] Chang SJ, Koh SB, Kang D, Kim SA, Kang MG, Lee CG, et al Developing an occupational stress scale for Korean employees. Korean J Occup Environ Med, 2005; 17:297–317.

[23] Sohn SI, Kim DH, Lee MY, Cho YW. The reliability and validity of the Korean version of the Pittsburgh Sleep Quality Index. Sleep Breath, 2012; 16(3):803–812. 10.1007/s11325-011-0579-9 21901299

[24] Buysse DJ, Reynolds CF, Monk TH, Berman SR, Kuper DJ. The Pittsburgh sleep quality index: a new instrument for psychiatric practice and research. Psychiatry Res, 1989; 28:193–213. 10.1016/0165-1781(89)90047-4 2748771

[25] Chandola T, Brunner E, Marmot M. Chronic stress at work and the metabolic syndrome: prospective study. BMJ, 2006; 332:521–525. 10.1136/bmj.38693.435301.80 16428252 PMC1388129

[26] Kawada T. Relationship between components of the metabolic syndrome and job strain using a brief job stress questionnaire (BJSQ). Int Arch Occup Environ Health, 2013; 86:725–726. 10.1007/s00420-013-0870-0 23584233

[27] Lee CD, Blair SN, Jackson AS. Cardiorespiratory fitness, body composition, and all-cause and cardiovascular disease mortality in men. Am J Clin Nutr, 1999; 69(3):373–380. 10.1093/ajcn/69.3.373 10075319

[28] Wessel TR, Arant CB, Olson MB, Johnson BD, Reis SE, Sharaf BL, et al Relationship of physical fitness vs. body mass index with coronary artery disease and cardiovascular events in women. JAMA, 2004; 292(10):1179–1187. 10.1001/jama.292.10.1179 15353530

[29] Yoon ES, Jae SY. Association of occupational stress and cardiorespiratory fitness with cardiovascular disease risk factors in office workers. Korean J Sports Med, 2017; 35(1):48–56. 10.5763/kjsm.2017.35.1.48

[30] Pavett CM, Butler M, Marcinik EJ, Hodgdon JA. Exercise as a buffer against organizational stress. Stress Med, 1987; 3(2):87–92. 10.1002/smi.2460030204

[31] Strohle A. Physical activity, exercise, depression and anxiety disorders. J Neural Transm, 2009; 116:777–784. 10.1007/s00702-008-0092-x 18726137

[32] Hellerstedt WL, Jeffery RW. The association of job strain and health behaviors in men and women. Int J Epidemiol, 1997; 26:575–583. 10.1093/ije/26.3.575 9222783

[33] Kouvonen A, Kivimaki M, Elovainio M, Virtanen M, Linna A, Vahtera J. Job strain and leisure-time physical activity in female and male public sector employees. Prev Med, 2005; 41:532–539. 10.1016/j.ypmed.2005.01.004 15917049

[34] Jeon HJ, Park SJ, Shin DH, Chung IS, Lee MY. The relationship between the Korean occupational stress scale and pulse wave velocity among male firefighters. Korean J Occup Environ Med, 2011; 23(4):450–462.

[35] Kong JO, Koh SB, Chang SJ, Cha BC, Chung HK, Choi HR, et al Relationship between job stress and pulse wave velocity as a cardiovascular risk factor. Korean J Occup Environ Med, 2004; 16(4):450–458.

[36] O’Neal DN, Dragicevic G, Rowley KG. A cross-sectional study of the effects of type 2 diabetes and other cardiovascular risk factors on structure and function of nonstenotic arteries of the lower limb. Diabetes Care, 2003; 26:199–205. 10.2337/diacare.26.1.199 12502681

[37] Cohen H., Kotler M., Matar M.A., Kaplan Z., Miodownik H., Cassuto Y. Power spectral analysis of HR variability in post-traumatic stress disorder patients. Biol Psychiatry, 1997; 41:627–629. 10.1016/s0006-3223(96)00525-2 9046997

[38] Sloan RP, Shapiro PA, Bagiella E, Boni SM, Paik M, Bigger JT Jr, et al Effect of mental stress throughout the day on cardiac autonomic control. Biol Psychol, 1994; 37:89–99. 10.1016/0301-0511(94)90024-8 8003592

[39] Vgontzas AN. Chronic insomnia is associated with nyctohemeral activation of the hypothalamic-pituitary-adrenal axis: clinical implications. J Clin Endocrinol Metab, 2001; 86(8):3787–3794. 10.1210/jcem.86.8.7778 11502812

[40] Wesenstein NJ, Balkin TJ, Belenky G. Does sleep fragmentation impact recuperation? A review and reanalysis. J Sleep Res, 1999; 8:237–245. 10.1046/j.1365-2869.1999.00161.x 10646163

[41] Ikehara S, Iso H, Date C, Kikuchi S, Watanabe Y, Wada Y, et al Association of sleep duration with mortality from cardiovascular disease and other causes for Japanese men and women: the JACC study. Sleep, 2009; 32:295–301. 10.1093/sleep/32.3.295 19294949 PMC2647783

[42] Curcio G, Ferrara M, Gennaro LD. Sleep loss, learning capacity and academic performance. Sleep Med Rev, 2006; 10(5):323–337. 10.1016/j.smrv.2005.11.001 16564189

[43] Partinen M. Sleep disorders and stress. J Psychosom Res, 1994; 38(1):89–91. 10.1016/0022-3999(94)90139-2 7799255

[44] Kim HC, Kwon KS, Koh DH, Leem JH, Park SG, Shin JY, et al The relationship between job stress and psychosocial stress among nurses at a university hospital. Korean J Occup Environ Med, 2006; 18(1):25–34.

